# The Effects of the Medium of Notetaking on the Delayed Learning Effect of College Students: A Mediated Moderation Model

**DOI:** 10.3390/bs14090756

**Published:** 2024-08-27

**Authors:** Lei Xu, Shuangshuang Cai, Yanxi Liu, Jiwen Chen, Shun Peng, Frank Andrasik

**Affiliations:** 1 School of Education, Jianghan University, Wuhan 430056, China; xulei@jhun.edu.cn (L.X.); shuangshuang.cai@jhun.edu.cn (S.C.); 15717189153@163.com (Y.L.); pengshun@jhun.edu.cn (S.P.); 2 Department of Psychology, University of Memphis, Memphis, TN 38152, USA

**Keywords:** college students, medium of notes, delayed learning effect, review, word count

## Abstract

This study systematically probed the relationship between the medium of taking classroom notes (virtual variable, electronic notetaking = 0 vs. traditional notetaking = 1), the word count in each medium, as well as the review process, and the students’ delayed learning effect for each notetaking approach. Data were collected from 189 college students, with the influence of gender and prior knowledge being controlled. The conclusions were as follows. (1) The notetaking medium was positively correlated with delayed test scores, irrespective of whether reviews were allowed or not. (2) The mediating role of word count between notetaking medium and delayed test scores was moderated by review. That is, when reviews were allowed, a significant correlation was found between the medium of the notes and the delayed test scores; when reviews were not allowed, the mediating effect of word count was not significant.

## 1. Introduction

Scientific and technological progress is undoubtedly one of the most significant driving forces for the profound change in contemporary education ecology. With the rapid development of information technology, the field of education is undergoing an unprecedented digital transformation. Electronic devices have become an indispensable part of individual learning. Electronic notetaking, defined as “the act of using Microsoft and related Office software to take notes in electronic devices” [1] has gradually become a part of people’s learning lives. Many students tend to type notes on computers instead of taking notes by hand [2,3]. Therefore, what impact the change in notetaking media will have on student learning has become an urgent question to answer.

Although some studies have pointed out that the “pen is mightier than the keyboard”, advances in electronics are turning the generative processing of notetaking into nongenerative transcription [4]. However, other researchers believe that taking notes on a computer is more conducive to learning [5]. When such contradictions arise across studies, it seems reasonable to show caution and not tout one notetaking medium over the other [6]. To explore the impact of the two notetaking mediums on learning performance, we need to start with notetaking strategies, which includes both encoding and external storage functions. According to the tenants of distributed memory, the encoding function that occurs during the act of notetaking benefits both learning and retention, thus improving internal memory. However, the external storage function emphasizes the importance of utilizing notes to store information to facilitate the subsequent reviewing of material, thus enhancing external memory [7,8]. Electronic notetaking, which turns the traditional paper storage of handwriting into more fluent electronic digital copies of writing, allows for higher storage efficiency and consequent lower search costs. However, over-reliance on storage function may come at the cost of deeper encoding benefits, a finding supported by Mueller and Oppenheimer [4]. In their study, all undergraduates were instructed to take notes through a designated medium while watching a TED talk, after which they were immediately tested. Participants who took electronic notes typed significantly more words than those who took notes by hand, with the word count positively predicting their immediate recall performance. Furthermore, students who used laptops for notetaking revealed more verbatim overlap than did their longhand counterparts, with the verbatim overlap shown to negatively predict their study performances. It is worth noting that the effect of verbatim overlap on test scores was stronger than that of word count.

However, the enhanced effects on encoding and external-storage functions are most often evident (or found) when learners all allowed to review their prepared notes [4]. Bui and his team found [9] that when individuals were not allowed to review, verbatim-notetakers scored significantly lower on a delayed test when compared to an immediate test, while notetakers who organizationally paraphrased the learning material revealed similar levels of enhanced retention at both testing intervals. In other words, in a review-absent situation, the delayed study effect for electronic notes was also inferior to that for traditional notetaking. This is because the favorable encoding effect of electronic notes caused by recording more was weakened by forgetting in the delay test, while the disadvantage in the immediate test due to insufficient generative processing was maintained. Hence, people rely more on review, anticipating that the stronger storage of electronic notes can neutralize its deficits in encoding. This critical question determines how we should assess the severity of the insufficient encoding capacity of electronic notes. Accordingly, it is necessary to deeply explore how the notetaking medium affects the delayed study performance from the perspective of review or no review.

Review promotes the interaction of new and old knowledge by arousing the recognition of the outside-stored information. In essence, it is a constructive process between prior knowledge encoded before review and learning material in notes [10]. Review outcomes are closely related to prior knowledge and learning material and are a trade-off between the quantity and quality of notes. Whether learning material can be better constructed depends on the prior knowledge. Review could improve the retention of the encoding effect for transcription notetakers [11]. One research work has shown that when review is allowed, word count is positively correlated with delayed test performance. Therefore, more notes were undeniably advantageous to review, and more transcribed notes would be more beneficial when review is allowed [9]. However, another research work has argued that more notes recorded by laptops were insufficient to offset its disadvantages in cognitive processing [4]. Alexa’s [12] research showed that compared with electronic notes, paper-and-pencil notes were more beneficial for long-term memory as the number of reviews increased. 

It can be seen from the above discussion that the effect of electronic notetaking on delayed learning is still controversial. One thing is certain, however: that review makes a difference. So, we designed this study to examine the moderating effect of review. Accordingly, we propose hypothesis 1, that when review is not allowed, the notetaking medium (virtual variable, electronic notes = 0, traditional notes = 1) will positively predict the delayed test scores, with no significant mediation effect of word count. We proceed with hypothesis 2, that when review is allowed, the notetaking medium is positively correlated with delayed learning performances. Meanwhile, the word count will significantly mediate the relationship between the notetaking medium and the delayed test scores, as notetaking medium negatively predicts delayed test performance through word count.

Overall, we aimed to explore whether review would moderate the mediating effect of word count on the notetaking medium and the delayed test performance under the control of extra variables (i.e., undergraduates’ prior knowledge, attitudes towards electronic notes, gender; Reddington) [4].

## 2. Materials and Methods

### 2.1. Participants

As a field study, four public elective classes, including psychology, electrical mechanics, computer science, and other majors, were randomly cluster sampled from two universities (two classes per university, so that the level of universities and majors was matched) in China. Two classes in the same university (at time 1) were randomly assigned to an experimental condition: the electronic-notetaking or traditional-notetaking group. Then (at time 2), half of the students in each class were randomly assigned to an experimental condition: the review-present or review-absent group. Yet, regrettably, due to the random cluster sampling method forced by the real environment, we only achieved this by matching different levels of universities and majors. Other demographic variables, such as gender, can only be controlled for by statistical means.

All 236 participants were proficient in operating their computers and office software. After screening out invalid data, such as those who omitted any items (n = 45), and excluding 2 participants who dropped out of this study, 189 participants (80% of those initially enrolled) remained for analysis. The distribution was as follows: 89 valid cases completed their participation in the electronic-notetaking group (38 for the review-present group, where there were 23 males and 15 females; 51 for the review-absent group, comprising 7 males and 44 females), and 100 in the traditional-notetaking group (60 for the review-present group, where there were 18 males and 42 females; 40 for the review-absent group, comprising 5 males and 35 females).

### 2.2. Material and Measurements

Learning Material. We downloaded a video clip titled The Palace Museum from the Sohu Video website as our stimulus. The transcript of the video was the text of a Chinese textbook of the China People’s Education Press for grade eight, lesson 14 in the first volume. The amount of text was 1600 words. To ensure that the participants had enough time to take notes, the original video was slowed down from 8 min and 20 s to 16 min and 34 s.

Measurements of Control variables. Considering that the prior-knowledge level and attitude towards electronic notes will affect the follow-up learning effect, these were measured as the control variables for the follow-up analysis.

Prior-Knowledge Level: To ensure content validity, a prior-knowledge test was developed by five educational psychologists jointly, drawing upon common-sense issues about the Palace Museum, which included 10 Yes/No and 11 fill-in-the-blank questions. One point was scored for each question, resulting in the highest personal total score of 21. 

Attitudes towards Electronic Notes: Following Seigel’s approach [13], attitudes were tested with one item on a 5-point Likert scale: “What is your attitude towards using electronic means (such as computer, mobile phone, etc.) to take notes while studying?”.

Measurements of dependent variable. The Delayed Test Score: A delayed test was developed by the same five educational psychologists, which contained 21 fill-in-the-blank questions, worth one point for each question. The personal total score was between 1 and 21. 

Measurements of mediate variable. The Word Count: The number of words in the traditional-notetaking group was calculated manually, while this was performed automatically in the electronic-notetaking group by the office software. 

### 2.3. Procedure

At time 1, the students were first asked to complete the prior-knowledge level test and the demographic scale. The video of the learning material was then played, and students in both the longhand and laptop groups were instructed to use traditional-notetaking and electronic-notetaking strategies, respectively, after which their notes were collected. After one week (time 2), participants (who were not informed that a follow up test would be included) at time 1 were required to complete the delayed test. Before the test, the review-present group was allowed to take back their notes and review them for five minutes, while the review-absent group was solely allowed to “mind review”, without access to their notes, for five minutes. 

## 3. Results

### 3.1. Descriptive Analysis

The scores of study variables in different situations are shown in Table 1.

### 3.2. Correlation Analysis

As shown in Table 2, the medium for notetaking was correlated positively with the delayed test scores and negatively with the word count in both groups. In addition, the word count positively predicted the delayed test scores. This means that laptop typists can take more notes, and more notes indeed can be beneficial to the learning results to some degree. However, whether review was permitted or not, electronic notes were generally inferior to the traditional notes in terms of the delayed test scores. Moreover, these results also provided preliminary support for further mediation and moderation analyses. 

### 3.3. Mediation and Moderation Analyses

Because the notetaking medium is a virtual variable and review is a categorical variable, structural equation modeling (SEM) multigroup analysis was used to examine the moderation effect of review on the mediation effect of the word count [9,14,15]. The underlying idea is that when the mediation effect is significant in one group but not in another group, this effect may be moderated by the grouping variable. 

**Figure 1:**
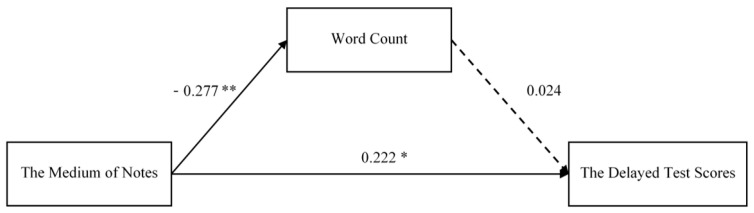
Review-absent group: the mediation effect of the word count. * *p* < 0.05, ** *p* < 0.01. Dotted line: the path coefficient is not significant.

Firstly, we examined whether the word count mediated the relationship between the notetaking medium and the delayed test scores in the review-absent group. After controlling for gender and the attitude to electronic notes, we used the Mplus 7.0 maximum likelihood ratio test to build the model. Third, we tested the mediation hypotheses using a bias-corrected bootstrap 95% confidence interval analysis based on 5000 bootstrap samples [16]. SEM yielded a moderate model fit: *χ*^2^ = 6.180, *df* = 5, *p* > 0.05; *RMSEA* = 0.051, *CFI* = 0.967, *TLI* = 0.941, *SRMR* = 0.051. As shown in Figure 1, there was a positive direct effect between word count and delayed test scores, while its path coefficient was not significant. The data in Table 3 further show that the indirect effect of the word count on bootstrap 95% confidence intervals contains a value of 0, so that in the review-absent group, word count did not significantly mediate the relationship between notetaking medium and delayed test scores. Therefore, hypothesis 1 was supported. 

**Figure 2:**
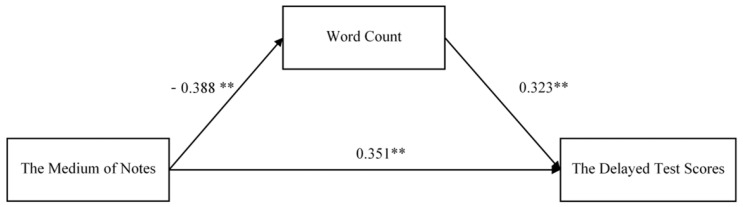
Review-present group: the mediation effect of the word count. ** p < 0.01.

Next, we examined the mediation effect of word count in the review-present group. SEM also yielded a moderate model fit: *χ*^2^ = 0.865, *df* = 1, *p* > 0.05; *RMSEA* = 0.000, *CFI* = 1.000, *TLI* = 1.031, *SRMR* = 0.017. As shown in Figure 2, there was a positive direct effect between word count and delayed test scores. The notetaking medium indirectly negatively predicted the delayed test scores via the word count. According to Table 3, a value of 0 was not contained in the indirect effect of the word count on bootstrap 95% confidence intervals, suggesting that word count partially mediated the relationship between the medium and the scores. The ratio of absolute values of both indirect and direct effects was 35.7%. Thereby, hypothesis 2 was supported. 

To sum up, the partial mediation effect was found only in the review-present group, so that review moderated the mediation effect of the word account on the notetaking medium and the delayed test scores. Thus, whether review is allowed or not, the pen is mightier than the keyboard. Though the use of laptops facilitates the verbatim transcription of video content, it has less advantages for delayed test scores when reviewing is forbidden. Accordingly, taking more notes benefits reviewing to some extent, but this is not enough to offset its deficits in encoding due to the tendency to make thoughtless transcriptions, occasioned by taking electronic notes. 

## 4. Discussion

### 4.1. Relationship between Notetaking Medium and Delayed Learning Effect under Review-Absent Group

The current study found that electronic notes predicted poorer performance than traditional notetaking in the delayed test. When review was not allowed, notetaking medium was positively related to delayed test scores, while no significant mediation effect of word count was found. 

The levels of the processing framework suggest that organized notetaking (i.e., summarizing, concept mapping) involves deeper cognitive processing and thus promotes enhanced information retention [17]. However, transcribed notes have generally been found to be less effective at promoting encoding. Their encoding function is mainly shown in the convention between orthographic and phonological processes when the learner records the input information, such as writing down what is heard [18]. The greater the number of notes taken, the more processing conversions will be generated. Though this benefits the immediate learning effect to some extent, it has been shown to impair retention, which means that delayed learning is less effective [19]. This was supported by our finding of there being no significant mediating effect of word count between notetaking medium and delayed test scores. Hence, when not allowed to review, the longhand participants also outperformed the laptop-typing participants in the delayed test. 

The results suggest that, compared with notetaking media, the level of notetaking processing seems to be more worthy of attention. Whether it is electronic notes or traditional notes being taken, it is essential to take organized notes of the learning material. The organized notetaking of learned material can combine the external storage and easy-search functions, as well as the encoding function of electronic notes.

### 4.2. Relationship between Notetaking Medium and Delayed Learning Effect under Review-Present Group

People rely on the notion that reviewing can partially offset the detriment associated with electronic notes to encoding. However, the current study casts doubt on this reliance. We empirically supported Mueller’s view that more notes being taken with electronic notes hardly balanced the associated deficits in encoding. In general, it showed that when reviewing was allowed, notetaking medium positively predicts delayed test performance, while word count negatively mediates this relationship. 

As mentioned, the essence of reviewing is to construct prior knowledge encoded before the review and learning the material in one’s notes [10]. Indeed, more learning material benefits learners to some degree. Consistent with previous studies, our findings also showed that when reviewing is allowed, notetaking medium negatively predicts delayed test performance through word count. Yet, prior knowledge is the basis of whether more learning material can engender more constructive processing. To take an extreme example, reviewing is unhelpful when all coding effects before reviewing are forgotten. This is because all notes will be newly and thoroughly learned. This was supported by our findings that the positive direct effect between notetaking medium and delayed test scores was weaker than the negative indirect effect of the word count. 

It is worth noting the studies in which note integrity promotes performance. Given that the true benefit of lecture notetaking appears to stem from the external storage function [20], it is plausible that the extent to which students record incomplete lecture ideas into their notes hinders the external storage function of lecture notes and diminishes learning and achievement. Regardless of notetaking medium, however, participants failed to record most of the ideas presented during the lesson on average, recording just 35% of lesson ideas, and 20% of those noted ideas were partial notes [21]. Despite the speed advantage of typing notes, computer-using notetakers still record fewer than half of the ideas presented during lectures [2,9,22]. As Morehead points out, most college students are not sufficiently trained in the use of electronic notetaking [2]. However, there is no doubt that electronic notetaking as a technology requires adequate training. If a digital-note notetaker wanted to take notes in a certain way but their word processing software was not pre-set to do so, they would need to search for and adjust the settings during the notetaking process, thereby theoretically diverting attention to software settings rather than attending to the incoming speech [13]. Therefore, we believe that the advantages of electronic notes are not fully represented in this study. When the electronic-note notetaker is given more time to train, the distraction caused by this lack of proficiency will be reduced, more attention will be transferred to the content of the lecture, the completeness of the notes will be increased, and the effect of reviewing will be enhanced.

### 4.3. Brief Summary

Firstly, this study affirms the positive role of reviewing in the learning process. The true value of notetaking is more in the review of notes than in their recording [23]. Other researchers have also found the activity of notetaking cognitively demanding and sometimes ineffective when recorded notes are not reviewed [24]. Review makes the external storage function of electronic notes realized.

Secondly, notetaking is not only a way for students to learn and understand knowledge [25]. For teachers, it is also a signal that students are paying attention [26]. Researchers have shown that electronic notetaking can improve student performance in classroom settings [27] and that “deliberate engagement” in large classes can increase student engagement. Moreover, this strong interaction with the teacher and high level of activity in the class is also reflected in communication over the Internet: emails and social media are often distractions for electronic notetakers [28], as well as for other people sitting nearby [13]. In addition, one study has shown that 20% of students now experience digital distraction compared to when they did not use electronic devices in class [29]. Therefore, in addition to the impact of the storage and encoding function of electronic notes on academic performance, its role and influence in the process of learning is also worthy of further in-depth study.

Lastly, having diverse options is the most important feature of self-regulated learning [11]. Similarly, when taking notes, learners can also select what information to record or to neglect, and it is the content of the information rather than how much information is written down that is most important. Among all kinds of note symbols, pictures play an important role. Some studies have combined pictures and notes to effectively promote students’ learning [30]. However, compared to electronic notes, pens and paper are viewed as providing a less restrictive format, with symbols, diagrams and graphs easier to draw by hand [31]. Research has found that longhand notetakers did record more images in their notes while the lesson was ongoing than computer-note notetakers. Students need to be guided on how to recognize and record the value of course-related images [21].

### 4.4. Limitations and Future Research

Despite the rigorous design of this study, combined with previous research, there are still some shortcomings. (1) Due to the limitation of notetaking software, this study only examined the transcription of electronic notes. As mentioned above, organized notes are more conducive to realizing the positive effects of notes, so subsequent studies can pay more attention to organized notes in either notetaking medium. (2) The measurement of prior knowledge as the control variable, using a non-scale questionnaire, can only test its content validity, and more standardized measurement could be used in subsequent research. (3) The representativeness of the samples needs to be strengthened. In the sampling, statistical control was taken for demographic variables such as gender, limited by the conditions of the field study. Follow-up studies should carry out stricter controls to enhance the representativeness of the sample.

## 5. Conclusions

We conclude with that the following: (1) Whether a learner was allowed to review or not, the medium of notes was positively correlated with their delayed test scores. (2) After controlling for extraneous variables, reviewing moderated the mediating effect of the word count on the note medium and the delayed test scores. Specifically, under the review-absent condition, the mediating effect of the amount of text was not significant, while, under the review-present condition, the note medium impaired the delayed test scores through the amount of text. (3) Electronic notes have more external storage and faster search capabilities, but this does not make up for their encoding shortcomings during review. This study emphasizes that it is not the notetaking medium that is important, but rather how notetaking is organized, rather than transcribed. This suggests to educators that in the formative stage of notetaking, no matter what kind of notetaking medium is used, it is crucial to carry out productive/organizational notetaking. After taking notes, review plays an irreplaceable role. In addition, for the developers of electronic notetaking software, it is necessary to enrich the notetaking function of the software, especially in the recording of picture information, and to explore alternative note-transcription assistance software. For users of electronic notes, it is important to increase the proficiency of notetaking and to increase the awareness of the content of the notes.

## Figures and Tables

**Table 1:** Descriptive analysis of study variables in review-present and review-absent groups.

	Review-Absent (N = 91)				Review-Present (N = 98)			
	Electronic Notes (n = 51)		Traditional Notes (n = 40)		Electronic Notes (n = 38)		Traditional Notes (n = 60)	
	M	SD	M	SD	M	SD	M	SD
Word Count	374.10	157.02	301.13	89.98	285.13	133.16	214.38	103.08
Delayed Test Scores	7.65	3.67	9.33	3.23	9.37	3.61	11.15	2.79
Prior Knowledge Level	6.00	2.08	6.20	1.88	6.42	1.57	6.33	3.16
Attitudes to Electronic Notes	2.78	0.90	2.75	0.78	2.95	0.57	3.02	0.83

**Table 2:** Inter-correlations of study variables in review-present and review-absent groups.

	Review-Absent (N = 91)					Review-Present (N = 98)				
	1 (Medium of Notes)	2	3	4	5	1	2	3	4	5
2. Word Count	−0.27 *					−0.29 *				
3. Delayed Test Scores	0.24 *	−0.03				0.27 **	0.25 *			
4. Gender	0.02	−0.10	0.24 *			0.30 **	0.24 *	0.25 *		
5. Prior Knowledge Level	0.05	0.22 *	0.35 **	−0.07		−0.02	0.16	0.22 *	0.16	
6. Attitudes to Electronic Notes	−0.02	0.18	−0.29 **	−0.15	−0.09	0.05	0.16	0.15	0.21 *	0.34

* p < 0.05, ** p < 0.01, (medium of notes were virtual variables: electronic notes = 0, traditional notes = 1).

**Table 3:** Analysis of the mediation effect of the word count.

Groups	Effect Size	Boot Effect Error	BootCI Lower Bond	BootCI Upper Bond
Review-Present	−0.007	0.033	−0.070	0.057
Review-Absent	−0.125	0.043	−0.210	−0.041

## Data Availability

The datasets used and analyzed in the current study are available from the corresponding author, J.C. (chenjiwen@jhun.edu.cn), upon reasonable request.
